# Epidemiology of Hepatitis B and C Infections in Al-Anbar/Iraq and Correlation Between Viral Load and Liver Function

**DOI:** 10.1155/av/9970549

**Published:** 2025-06-26

**Authors:** Mohammed A. Hamad, Ala'a F. Habeeb, Nabaa A. Muhammed, Rawaa A. Muhammed

**Affiliations:** ^1^Biotechnology Department, College of Applied Sciences, University of Fallujah, Fallujah, Iraq; ^2^University of Fallujah, Fallujah, Iraq; ^3^Pathological Analysis Department, College of Applied Sciences, University of Fallujah, Fallujah, Iraq; ^4^Fallujah Teaching Hospital of Maternity and Children, Ministry of Health, Fallujah, Iraq

**Keywords:** HBV, HCV, incidence, liver function, q-PCR techniques, viral load

## Abstract

**Aims:** Hepatitis B (HBV) and C (HCV) represent a menacing health problem worldwide and its risk of contamination and transmission by routine activities and contact with infected patients and its remarkable adverse effects and presence of silent carriers. The diagnosis developed with the developing techniques using more specific techniques. The aim is to study the epidemiology and molecular detection of HBV and HCV in the Anbar governorate (Fallujah and Amiriyah) and the correlation between the viral load of HBV and HCV on liver functions.

**Materials and Methods:** A cohort of 5463 tested for HBV viral infection and 5873 tested for HBV viral infection patients' information were collected from units in Fallujah and Amiriyah Hospitals as well as private laboratories who sent for HBV or HCV detection from 15^th^ January 2021 to 20^th^ November 2021 using a questionnaire and diagnosed with rapid tests, the positive results subjected for testing with ELISA and samples tested using q-PCR techniques.

**Findings:** Among the studied cohort, the prevalence of HBV infections was high compared to HCV, with the highest impact in females and the ages of young adults (29–30 years). Furthermore, viral loads were high in HCV-infected patients compared to HBV. Finally, liver enzymes (ALP, AST, and ALT) were significantly high in HCV-infected patients compared to HBV.

**Conclusion:** The study concluded that females have a higher rate of infection with higher progression of the HBV and more morbid liver enzymes. The highest affected age groups are the highest communicational and economic activity group of young adults with elevated impact on the liver with HCV viral load increasing.

## 1. Introduction

Hepatitis viral infection represents menacing infections that spread globally, which cause chronic state and result in inflammation of the liver leading to elevated risk of morbidity and mortality [1]. Hepatitis is caused by among one from the total five types of viruses affecting the liver (A, B, C, D and E) [2]. Acute infections with B (HBV) and C hepatitis (HCV) may exacerbate to form cancer or cirrhosis of the liver; moreover, a severe contamination may exacerbate and lead to elevated bilirubin leading to jaundice [3] with disrupted liver functions indicated by elevated liver enzymes resulting from mild to severe inflammation with the possibility to become a chronic state mainly in infants (90%) in comparison to adults (2%–6%) [4]. Chronic HBV can lead to serious health issues, such as cirrhosis or liver cancer. 70%–85% of people who become infected with HCV form the chronic state of infection [5], which causes mainly long-term contraindications and may exacerbate to cause mortality when the majority of those cases initiate with silent transferable infection [6]. A mortality of more than 1 million people is caused by HBV infection and its complications. Recent studies in Iraq elucidated that the incidence of HBV and HCV were 0.78% and 0.2%, respectively [7, 8]. Many HBV infections come in combination with HCV leading to increasing morbidities including liver cirrhosis and hepatocellular carcinoma [8, 9].

Despite that, laboratory methods recruited for hepatitis virus diagnosis have pros and cons, as little information is provided by the researchers on comparing the results obtained from the different methods [10, 11]. Rapid immunochromatographic tests give reliable time efficiency and preliminary results to initiate virus infection diagnosing especially in the blood. Moreover, ELISA tests have more advanced sensitivity and specificity, while comparing both techniques with PCR procedure as the gold standard requires study, since this is a more costly method with a long time but good efficiency. Since the clinical laboratories depend mainly on the immunologic tests [12], the aim of the research is to study the epidemiology of HBV and HCV in Anbar governorate (Fallujah and Amiriyah) and risk factors in the population, in addition to studying the impact of viral load in infected patients with HBV and HCV with the liver enzymes levels of mainly alkaline phosphatase, aspartate aminotransferase (AST), and alanine aminotransferase (ALT).

## 2. Methods

A total cohort of 5463 tested for HBV viral infection and 5873 tested for HBV viral infection patient information were collected from patients tested either routinely before surgical or medical interventions or tested intentionally for both HBV and HCV infections (from Fallujah Teaching Hospital for Maternity, and Amiriyah Hospital, as well as private laboratories in Fallujah city during the period from 15^th^ January 2021 to 20^th^ November 2021). All participants were informed of the study objectives. The collected samples were registered for their ages and gender state, the samples were diagnosed with rapid chromatographic tests, and positive results were confirmed with ELISA. The positive results in both the recent techniques were tested for viral load using q-PCR to study the correlation with liver function tests. Samples were collected from each patient in a gel tube with a clot activator and centrifuged after complete clotting of the sample using a centrifuge (Hitech, Germany) at 3800 RPM/5 min and then preserved by refrigeration MEDIA, China.

### 2.1. Detection of the Viral Infection

#### 2.1.1. Rapid Chromatographic Tests

Serum samples were tested using rapid test kits including HBsAg rapid test strip provided by DRG (USA) and an Anti-HCV cassette rapid test provided by Linear Chemicals (Spain); the protocols were performed also according to the manufacturer's instructions and have an accuracy of about 99.6% for the HBsAg test and a relative sensitivity of about 99% and specificity of about 99.5% for the Anti-HCV test according to the manufactured companies instructions.

#### 2.1.2. ELISA Test (HBsAg ELISA and HCV Ab ELISA)

The test was conducted according to the protocol of the kit supported by CTK Biotech (USA) on serum samples which have a relative sensitivity and specificity of about 100% according to the manufacturer's instructions.

#### 2.1.3. Detection of HBV and HCV's Viral Concentration Using q-PCR

Serum samples were subjected to extraction protocol using RealLine Extraction 100 (BIORON Diagnostics GmbH, Germany) according to the instruction of the supplemented company as follows.

Samples along with a positive control (PC) sample (from a RealLine PCR kit component) and a negative control (NC) sample were used for the detection. 30 μL of the IC solution was added to each tube of specimens (100 μL), PC (70 μL of NC and 30 μL of PC), and NC (100 μL), and then 300 μL of the Lysis Reagent was added to each tube, vortexed for 10–15 s, and then vortexed with heat for 10 min at 65°C and 1300 rpm. 400 μL of the nucleic acid precipitation solution was added to each tube, vortexed for 10–15 s, and then allowed to stand for 3–5 min at 18–25°C, after centrifugation for 5 min at 13,000 rpm at room temperature. The supernatant would be removed, and 500 μL of Wash solution 1 was added to the sediment, vortexed, and then centrifuged by removing the supernatant and washing by 300 μL of Wash solution v2 and vortexed and centrifuged; finally, the supernatant was removed and pellet dried for 2–3 min at 18–25°С.

Quantitative detection of HBV DNA by q-PCR was performed after extracting the viral DNA from serum specimens and detection of viral load by quantitative real-time PCR using q-PCR–HBV supplemented by BIORON Diagnostics GmbH (Germany). While the quantitative detection of HCV RNA by q-PCR was performed after the extraction of the viral RNA, we performed viral load reaction using q-PCR–HCV provided by BIORON Diagnostics GmbH (Germany) as follows: 50 μL of the prepared samples and controls added to the master mix was ready to use and then placed in a thermal cycler device by using the following program:

  Stage 1: 50°С, 2 min
  Stage 2: 94°С, 1 min
  Stage 3: Measurement of fluorescence at 
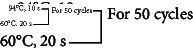

The FAM channel was used for the detection of the amplification of internal control DNA and the ROX channel for the detection of the amplification of HBV DNA or HCV RNA.

Ct value for PC must be in the range and NC should detect a FAM fluorescent signal increase and Ct value, and no significant ROX fluorescent increase should appear for starting the detection of sample result. The sample is considered negative if the Ct value via the ROX channel exceeds 40 or is not determined (the detailed manual for real-time PCR detection of HBV and HCV in the supporting file (available here)).

### 2.2. Liver Function Tests

All specimens were referred for quantification of HCV and HBV using q-PCR subjected for quantification of liver function enzymes including ALT/GPT and AST/GOT. The test was performed on the serum using kits provided by Roche Diagnostics (Germany) using a COBAS C111 device according to the manufacturer's instructions. The device must be calibrated to ensure the accuracy of test results.

### 2.3. Statistical Analysis

The Statistical Analysis System (SAS) (2012) program was used in this study to accomplish the *t*-test and Chi-square test under probability values (0.05 and 0.01).

## 3. Results

A total of 5463 patients tested for HBV viral infection and 5873 patients tested for HCV viral infection, where 66 patients (1.21%) out of 5463 patients were positive for HBV infection and 42 patients (0.72%) out of 5873 were positive for HCV. Females illustrated a highly significant (*p* ≤ 0.01) increase in the infection with both the hepatitis viruses (HBV and HCV) in the study group, where 42 cases of HBV and 30 cases of HCV were noticed in females, which is higher than the number of males infected with the both hepatitis viruses. Moreover, the number of cases infected with HBV in the study group is higher than HCV infection in both males and females, as illustrated in Table 1.

The highest age group infected with HBV (*p* ≤ 0.01) was 31–40 years, followed by 21–30 years age groups. Convergent results were obtained from the age groups infected with HCV, where the highest infected age group (*p* ≤ 0.01) was 21–30 years with 13 cases infected from the total 42 cases infected with HCV, as indicated in Table 2.

**Figure 1:**
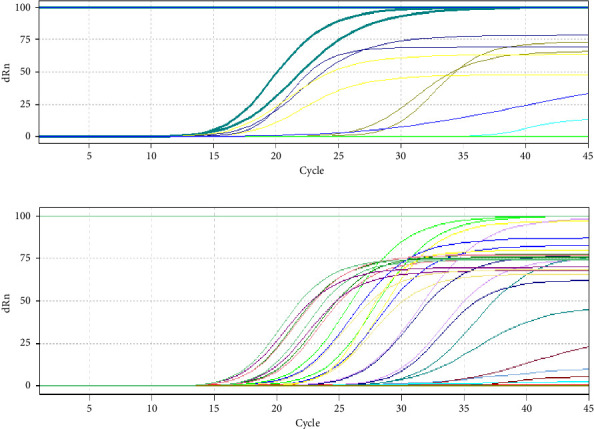
The results of Ct's in q-PCR for both HBV and HCV. (a) HCV curves in q-PCR. (b) HBV curves in q-PCR.

HBV viral load is significantly higher than HCV load as shown in Figure 1 and Table 3, despite that alkaline phosphatase enzyme concentration is higher in patients infected with HCV than HBV infection without showing any significant difference during statistical analysis; meanwhile, ALT showed significantly higher concentrations in HCV-infected patients than in HBV-infected ones (*p* ≤ 0.05). The highest significant difference between liver function enzymes compared between HCV and HBV was noticed in AST (*p* ≤ 0.01), where the concentration in HCV-infected patients was 97.16 ± 66.50 in comparison to HBV's 45.29 ± 16.74. This suggests a huge damage to the liver function caused by HCV in contrast to HBV. The results are shown in Table 3.

Females' viral concentration (despite regarding viral type: HCV or HBV) is significantly higher (*p* ≤ 0.01) than that of males. This is parallel to the findings of liver function tests, that illustrated higher values in females compared with males, Concentrations in females were 87.64 ± 20.38 U/L and 89.70 ± 10.25 U/L sequentially for ALT and ALP which is significantly higher than males (*p* ≤ 0.05). AST alone showed a significant increase in females (155.54 ± 95.16 U/L) under (*p* ≤ 0.01), as shown in Table 4.

## 4. Discussion

The HBV and HCV are challenging bloodborne diseases that represent a chronic infection with a high risk of transmission in the community and in healthcare practitioners [13]. According to the epidemiological information collected along the year, the study results revealed that there was a higher prevalence of HBV among the study groups compared to HCV infection in Fallujah and Amiriyah cities in 2021. This result was convergent with the recent studies and the established information from the references. However, it may be a confounding result when comparing those infections with the rate of HBV vaccination in the country and the present system for vaccination of all age groups especially in children. Even though a vaccine is available for HBV, both HBV and HCV infections are still a community health problem globally. In our study, the involved patients were sent to be checked for HBV and HCV infections accidentally due to their involvement in the blood donation program or they were referred as a result of presence of a specific clinical signs and symptoms. These samples were subjected to detection tests including rapid tests and ELISA by taking a survey about some information required for the questionnaire. As a result of the study survey, 66 cases out of 5397 and 42 out of 5331 showed positive results for HBV and HCV sequentially, where HBV incidence is higher than HCV, which agrees with the recent observations conducted by Mutlk et al. [8], who showed a high rate of HBV compared to HCV and represented a higher incidence in Al Ramadi Teaching Hospital and Al Ramadi Teaching Hospital for Maternity and Children than in our study in the same year; this may be due to the differences in the study group number, number of outpatients visiting those hospitals, and the hospital units involved. A constant or convergent rate was seen in a report presented by a WHO-supported study that the prevalence of HBV was higher than HCV in Iraq in 2006 [14], and a higher incidence rate appears in Khudhair et al.'s study [15]. Those infections spread mostly in the age group extended between 21 and 30 years in HCV and 31 and 40 years that converge mostly with Mutlk et al. [8], a study showing the highest rate of infections in the 25–50 years age group and the lowest cases in 51–60 years, with the absence of cases in the age groups higher than it. This is a different result from the conclusions of Mutlk et al. [8], a study that revealed the lowest incidence in 0–10 years with only 9 cases; this difference may be revealed by the grouping of samples into different age groups and the absence of age information of samples collected from Fallujah Teaching Hospital that deprive us from distribution of those samples in the age groups and may effect ages distribution. A study conducted by Raheem et al. [4] reported the same finding about the highest age group exposed to the infection (20–45 years) and reported that this age group is the economically and sexually active age group, the infection rates in Khudhair et al.'s [15] study were consistent with the present study, where the lowest infections were in ages higher than 60 years. Females are higher than males significantly in both types of hepatitis, which may be attributed to the high requirements for medical interventions in the hospital during pregnancy and delivery in the maternity hospital and the ability to transmit this infection to their neonates by maternal transmission; this result is supported by Israr et al. [16], a study which demonstrated the elevated incidence of HBsAg and HCV seropositivity in pregnant females undergoing blood transfusion recently. Our results disagree with other studies such as Mutlik et al.'s [8] study, who showed high incidence of males compared with females (53.5% and 46.5%, respectively) nonsignificantly, in addition to the results obtained by Tareq et al. [17] who reported the same finding in the percentage of male infections in results from dentist compared with females (14% and 10%, respectively). Moreover, Abdulwahab et al.'s [18] study reported the same results in haemodialysis patients. However, Raheem et al.'s [4] findings are contrary to our results, which reported a higher prevalence in females than males. The higher incidence in women is explained mainly according to the findings of Israr et al. [16]. In Majeed et al.'s study [19], among the 3914 pregnant women, 0.10% are infected with HCV, indicating a high prevalence in women as observed in our study. The recently indicated variance may be attributed to differences in the study population, sample collection method, and the techniques used in the diagnosis. Further studies on large cohorts from the same demographic population are required. HBV is ubiquitous in the liver; thus, the study aimed to assess the liver function related to the infection and correlated it with the viral load of hepatitis viruses. According to the results of our study, viral load of HCV-infected patients were higher than HBV load, which is related with high virulence of HCV, although the spreading and infectivity of HBV is the highest among hepatitis viruses. A considerable decrease in HCV infection rates may be caused by some prevention strategies and effective targeted therapies despite the absence of targeted vaccine [20]. On one side, mortality and morbidity due to HCV are not eradicated despite less incidence compared to HBV, and the burden of HCV-related liver disease is projected to increase in the next decade. A group of persons in the study were subjected to a liver function test by measuring the viral load using q-PCR and revealed a high amount of liver function enzymes (ALT, AST, and ALP) with a comparison between the two types of hepatitis viruses (HCV and HBV) that showed a higher amount of ALT and AST (significantly and nonsignificantly, respectively) in HCV than HBV in spite of having less amount of viral load of HCV. In comparison with Zaidan et al.'s [21] study, the results were consistent with our study, since they revealed a significant increase in serum transaminases enzyme (AST and ALT) in activities among patients with chronic hepatitis B. AST elevations often predominate in patients with cirrhosis and even in liver diseases that typically have an increased ALT [22], thus suggesting that this increase is synchronous with morbidity in the liver and transferring the status to the chronic state which is more common in HCV than HBV. While Raheem et al.'s [4] study showed no significant difference between HBV and HCV seropositivity and liver function markers in both males and females. In the study, females showed higher viral load and liver enzymes compared to males. Ages ranged from 18 to 71 years with a median equal to 51, and the most predominant age group was 31–51 years. Various potential mechanisms include the effect of sex hormones on oxidative and metabolic pathways, differential gene transcription in response to injury in women compared to men, and sex differences in immune regulation [23].

The study concluded that there was a higher prevalence of HBV infections in the study group than HCV in the study period significantly. Females have a higher rate of infection with hepatitis viruses than males and show higher progression of the HCV and more morbid liver enzymes. The highest affected age groups are the highest communicational and economic activity group of young adults (29–30 years) significantly. Viral load is higher in HCV-infected patients than in HBV-infected patients, and liver enzymes (ALP, AST and ALT) are higher in HCV-infected patients than in HBV-infected patients.

## 5. Conclusion

In conclusion, this research provides comprehensive data on the epidemiology and molecular insight of HBV and HCV in Al-Anbar Province particularly Fallujah and Amirah cities. According to the data, female patients had the highest rate of HCV and were suffering from liver dysfunction compared to males. These findings support the need for targeted screening programs in a population that appears to be at greater risk.

## Figures and Tables

**Table 1:** Distribution of the studied samples for HBV and HCV infection according to gender.

Type of virus	Gender	Positive no. (%)	Negative no. (%)	*p* value
HBV	Male (608)	24 (3.95%)	584 (96.05%)	0.0001^∗∗^
	Female (4855)	42 (0.87%)	4813 (99.13%)	
	Total (5463)	66 (1.21%)	5397 (98.79%)	0.0001^∗∗^

HCV	Male (515)	12 (2.33%)	503 (97.66%)	0.0001^∗∗^
	Female (5358)	30 (0.56%)	5328 (99.44%)	
	Total (5873)	42 (0.72%)	5331 (99.28%)	0.0001^∗∗^

^∗∗^ *p* ≤ 0.01.

**Table 2:** Distribution of the studied samples for HBV and HCV infection according to age.

Type of virus	Age group	Positive no. (%)	Negative no. (%)	*p* value
HBV	0–10	2 (3.03%)	280 (5.19%)	0.0001^∗∗^
	11–20	6 (9.09%)	505 (9.54%)	
	21–30	11 (16.67%)	711 (13.17%)	
	31–40	13 (19.70%)	395 (7.32%)	
	41–50	3 (4.55%)	115 (2.13%)	
	51–60	1 (1.52%)	63 (1.17%)	
	61–70	0 (0.00%)	161 (2.98%)	
	71–80	0 (0.00%)	111 (2.06%)	
	Fallujah Hos.	40 (1.29%)	3056 (98.71%)	0.0001^∗∗^
	Total (5463)	66 (1.21%)	5397 (98.79%)	0.0001^∗∗^

HCV	0–10	8 (19.05%)	280 (5.25%)	0.0001^∗∗^
	11–20	1 (2.38%)	505 (9.47%)	
	21–30	13 (3.09%)	711 (13.34%)	
	31–40	7 (16.67)	351 (6.58%)	
	41–50	0 (0.00%)	508 (9.53%)	
	51–60	1 (2.38%)	152 (2.85%)	
	61–70	0 (0.00%)	80 (1.50%)	
	71–80	0 (0.00%)	110 (2.06%)	
	Fallujah Hos.	12 (0.38%)	3152 (99.62%)	0.0001^∗∗^
	Total (5873)	42 (0.72%)	5331 (99.28%)	0.0001^∗∗^

^∗∗^ *p* ≤ 0.01.

**Table 3:** Comparison between HBV and HCV in hepatic function enzymes and correlation with viral load.

Type of virus	Mean ± SE			
	Virus concentration (IU/mL) (mean ∓ SD)	ALT (IU/mL) (mean ∓ SD)	AST (IU/mL) (mean ∓ SD)	ALP (IU/mL) (mean ∓ SD)
HBV	557,362.42 ± 228,870.93	45.29 ± 16.74	44.74 ± 16.11	74.35 ± 8.89
HCV	4,700,000.0 ± 2,300,000.0	97.16 ± 66.50	257.16 ± 118.68	76.30 ± 7.70
*t*-test	26,177.02^∗^	37.569^∗^	102.405^∗∗^	46.392 NS
*p* value	0.0170	0.0274	0.00409	0.927

Abbreviation: NS, nonsignificant.

^∗^ *p* ≤ 0.05.

^∗∗^ *p* ≤ 0.01.

**Table 4:** Effect of Gender and Type of virus in parameters study.

Gender	Virus concentration (IU/mL) (Mean ± SD)	Mean ± SE		
		ALT (U/L) (mean ± SD)	AST (U/L) (mean ± SD)	ALP (U/L) (mean ± SD)
Male	890,440.33 ± 563,043.22	31.21 ± 5.46	30.91 ± 3.89	63.94 ± 8.72
Female	1,614,877.20 ± 1,596,343.65	87.64 ± 20.38	155.54 ± 95.16	89.70 ± 10.25
*T*-test	271,392.05^∗∗^	42.36^∗^	72.361^∗∗^	20.026^∗^
*p* value	0.0001	0.02117	0.0034	0.0458

^∗^ *p* ≤ 0.05.

^∗∗^ *p* ≤ 0.01.

## Data Availability

The data that support the findings of this study are available on request from the corresponding author. The data are not publicly available due to privacy or ethical restrictions.

## Supporting Information

The supporting information in this article is provided in the “supporting information for production.”
