# Supplementary material for: Epidemiology of Hepatitis B and C Infections in Al-Anbar/Iraq and Correlation Between Viral Load and Liver Function
Source: Adv Virol. 2025 Jun 26;2025:9970549. doi: 10.1155/av/9970549 (PMC12226165; doi:10.1155/av/9970549)
Supplement: Supporting Information — Additional supporting information can be found online in the Supporting Information section. [file 9970549.f1.docx]

**Detection of HBV and HCV viral concentration using q-PCR:**

**Quantitative detection of hepatitis B virus (HBV) DNA by q-PCR.**

**- sample collection.**

Blood, serum or plasma (by EDTA or sodium citrate) specimens were used for extraction of viral DNA extracted using RealLine Extraction 100 (BIORON Diagnostics GmbH, Germany).

**- PROCEDURE PROTOCOL**

1. 50 μl of the corresponding extracted DNA solution was added to each tube using a separate pipette tip with filter. The reaction tubes placed into the thermal block of real time PCR device.

2. Program real time PCR device as follows:

Stage 1: 50°С, 2 min ;

Stage 2: 94°С, 1 min;

Stage 3: 94°C, 10 sec

50 cycles

60°C, 20 sec The measurement of fluorescence at 60 °С.

3. the selected amplification detection channel is FAM which used to collect Real-time PCR data for detection of amplification of **IC DNA,**  moreover; the channel **ROX** used for detection of amplification of **HBV DNA.**

**- Data Analysis**

**1.** In **Positive Control PC** sample and **Weak Positive Control WPC** sample (also for CS1 and CS2) the program should detect:

• **ROX** fluorescent signal increase and **Сt** value (HBV DNA amplification);

• **FAM** fluorescent signal increase and **Сt** value (IC DNA amplification).

**2. In Negative Control NC** sample the program should detect a **FAM** fluorescent signal increase and **Ct** value, and no significant **ROX** fluorescent increase should appear. If **Ct** value for NC along **ROX** channel is less than 40, this indicates the presence of contamination.

**3.** The program should detect amplification signal increase for IC DNA (channel **FAM**) in each sample and define **Ct** for IC. Probe analysis is valid if **Ct** of IC for this sample is equal to or less than 40.

**4.** Calculate (IC Сt)av as an average IC Сt of all specimens (including PC, WPC and NC). IC Ct values that differ by more than 2 from (IC Ct)av should be ignored. Recalculate (IC Ct)av for the remaining values.

**5.** The specimen is considered as **positive**, i.e. containing **HBV DNA**, if the Ct through **ROX** channel **is less than or equal to 40.**

**6.** The specimen is considered as **negative**, if the Ct through **ROX** channel is **above 40** or is not determined.

If **IC Ct** value for such sample differs from (IC Ct)av value by more than 2, the result is regarded as equivocal. A repeated analysis of the sample, starting with the DNA extraction step is required.

**7.** If the Ct value for NC through the **ROX** channel is **less than or equal to 40**, it indicates the presence of contamination. In case of contamination all positive results of this individual PCR test run are considered as equivocal. Actions are required to identify and eliminate the source of contamination, and repeat the analysis of all samples of this run that were identified positive. Samples that showed negative results in this run should be considered as negative.

8. If the calculated HBV DNA concentration is **higher than 108 IU/ml** should be interpreted as **positive with HBV DNA concentration “higher than 108 IU/specimen”.**

**9.** If the calculated **CMV DNA** concentration is less than 100 IU/ml, the result should be interpreted as **positive with HBV DNA concentration “less than 100 IU/specimen”**

10. Specimen is considered **negative** (not containing HBV DNA) when Ct via **ROX** channel for such specimen is **above 40 or is not determined**.

**Quantitative detection of hepatitis C virus (HCV) RNA by qRT-PCR.**

**- sample collection.**

Serum or plasma (by EDTA or sodium citrate) specimens were used for extraction of viral RNA extracted using RealLine Extraction 100 (BIORON Diagnostics GmbH, Germany).

**- Procedure Protocol**

1. Add **50 μl** of corresponding isolated RNA solution to each tube using a separate pipette tip with filter.

2. Place the tubes into the thermal block of real time PCR device.

**3.** Program real time PCR device as follows

Stage 1: 45°С, 30 min;

Stage 2: 94°С, 1 min;

Stage 3: 94°C, 10 sec

50 cycles

60°C, 20 sec

* measurement of fluorescence at 60 °С.

**4.**the selected amplification detection channel is FAM which used to collect Real-time PCR data for detection of amplification of **IC DNA.**

• moreover; the channel **ROX** used for detection of amplification of **HCV RNA.**

**- Data Analysis**

1. In **Positive Control** sample and **Weak Positive Control** sample (also for **CS1** and **CS2**) the program should detect:

• **ROX** fluorescent signal increase and Сt value (HCV cDNA amplification);

• **FAM** fluorescent signal increase and Сt value (IC cDNA amplification).

•The results of the individual PCR run are subject to analysis and accounting when HCV Ct value for PC is in the range specified in the insert for kits of this lot.

1. In **Negative Control** sample the program should detect a **FAM** fluorescent signal increase and **Ct** value, and no significant **ROX** fluorescent increase should appear.

3. If **Ct** value for NC along **ROX** channel is less than 40, this indicates the presence of contamination.

- The program should detect an amplification signal increase for IC cDNA (channel **FAM**) in each sample and define **Ct** for IC. Probe analysis is valid if **Ct** of IC for this sample is equal to or less than 40.

- Calculate (IC **Ct**)m as the average **Ct** value of IC for all samples (including PC and NC).

- Samples with **Ct** of IC, that differ from (IC **Ct**)m by more than 2, should be ignored. After screening, recalculate (IC **Ct**)m for remaining samples.

The sample is considered **negative** if **Ct** value via the **ROX** channel **exceeds 40 or is not determined**.

If **Ct** of IC for this sample differs from (IC **Ct**)m by more than 2, then result for this sample should be considered as equivocal. The test should be repeated from the sample RNA extraction stage.

The sample is considered **positive** if **Ct** value via the **ROX** channel **does not exceed 40**. If **Ct** of IC for this sample differs from (IC **Ct**)m more then 2, the sample is considered as positive without quantitative analysis. For quantitative analysis, repeat the test beginning from the RNA isolation stage.

If IC Ct value for such sample differs from the (IC Ct)av value by more than 2, then the sample is considered positive without stating RNA concentration. For quantitative analysis, it is necessary to repeat the analysis of the sample, starting with the isolation step.

If HCV Ct for WPC is above 40 or not determined, all negative results are considered invalid and require a repeated analysis
